# Supplementary material for: The CDK-PLK1 axis targets the DNA damage checkpoint sensor protein RAD9 to promote cell proliferation and tolerance to genotoxic stress
Source: eLife. 2017 Dec 19;6:e29953. doi: 10.7554/eLife.29953 (PMC5736350; doi:10.7554/eLife.29953)
Supplement: Supplementary file 1. — Primers used in this study was listed. [file elife-29953-supp1.docx]

**Supplementary File 1: *Primers used in this study***

| #1663: HsRad9_890F_NdeI | ATACCCATATGTCAGACACCGACTCGCACTCC |
| --- | --- |
| #1664: HsRad9_C_RBamHI | AATCTGGATCCTCAGCCTTCACCCTCACTGTC |
| #1686: HsRad9_S328_386AF | CCCTTGCACCTGGCCCCCAGCCCCCCAAGGGCCCCGGTCC |
| #1687: HsRad9_S328_386AR | GGGCCCTTGGGGGGCTGGGGGCCAGGTGCAAGGGAAATGG |
| #1688: HsRad9_S277AF | CCAGGACCTGGGCGCCCCAGAGCGTCACC |
| #1689: HsRad9_S277AR | CGCTCTGGGGCGCCCAGGTCCTGGGAGTGC |
| #1707: HsRad9_ST291_2_AF | CTCCAGGCTCACGCCGCACCCCACCCGGACG |
| #1708: HsRad9_ST291_2_A | CCGGGTGGGGTGCGGCGTGAGCCTGGAGCTGAGG |
| #2015: HsRad9_292AF | CTCCAGGCTCACAGCGCACCCCACCCGGACG |
| #2016: HsRad9_292AR | CCGGGTGGGGTGCGCTGTGAGCCTGGAGCTG |
| #2147: siRad9-resi1R | ATCAAAATGGATCGAAAGATTCAAGTTTGC |
| #2148: siRad9-resi1F | GAATCTTTCGATCCATTTTGATGCTCCAGG |
| #2149: siRad9-resi2F | CAGCAAATCTGAACCTGTCGATCCATTTTG |
| #2150: siRad9-resi2R | ATGGATCGACAGGTTCAGATTTGCTGACTC |
| #2452: plk400F_NdeI_pGADT7 | CGTACCAGATTACGCTCATATGAGGCAAGAGGAGGCTGAGG |
| #2453: plk600R_BamHI_pGADT7 | GCAGCTCGAGCTCGATGGATCCTTAGGAGGCCTTGAGACG |
| #2607:Plk2_ADF | GTACCAGATTACGCTCATATGGATTTCCACTTATCAAGCCCAGC |
| #2608: Plk2_ADR | CTGCAGCTCGAGCTCGATGTCAGTTACATCTTTGTAAGAGCATG |
| #2595: HsPlk3_polo_ADF | CGTACCAGATTACGCTCATCCCCCCAACCCAGCTAGGAGTC |
| #2596: HsPlk3_polo_ADR | GCAGCTCGAGCTCGATGGCTAGGCTGGGCTGCGGTCCCGGAGC |
| #2611: Plk4_ADF | GTACCAGATTACGCTCATATGAAATATATGACTGCACTTCACAG |
| #2612: Plk4_ADR | CTGCAGCTCGAGCTCGATGTCAATGAAAATTAGGAGTCGGATTAG |
| #2599: HsPlk5_polo_ADF | GTACCAGATTACGCTCATATGGCCTCACCCCTGTCGGAGATGTAC |
| #2600: HsPlk5_polo_ADR | GCAGCTCGAGCTCGATGGTCTACCATGCAGGGGTCCACTCTGACC |
