# Supplementary figures and images for: The CDK-PLK1 axis targets the DNA damage checkpoint sensor protein RAD9 to promote cell proliferation and tolerance to genotoxic stress
Source: eLife. 2017 Dec 19;6:e29953. doi: 10.7554/eLife.29953 (PMC5736350; doi:10.7554/eLife.29953)

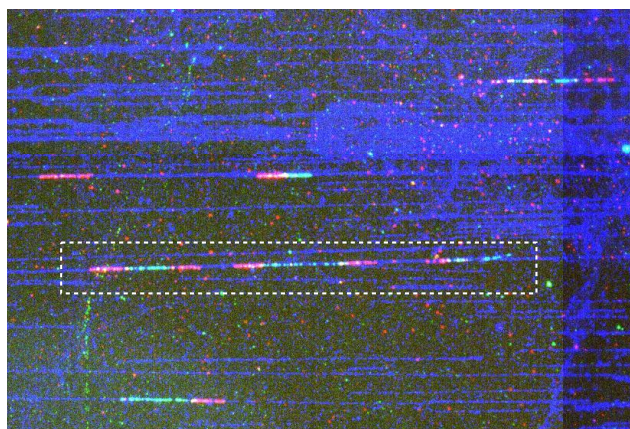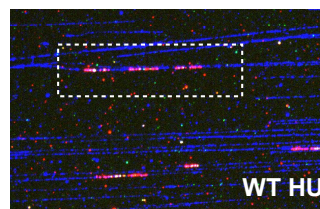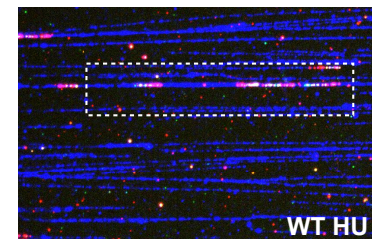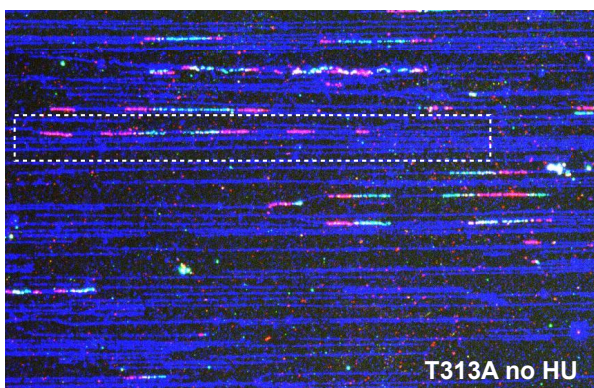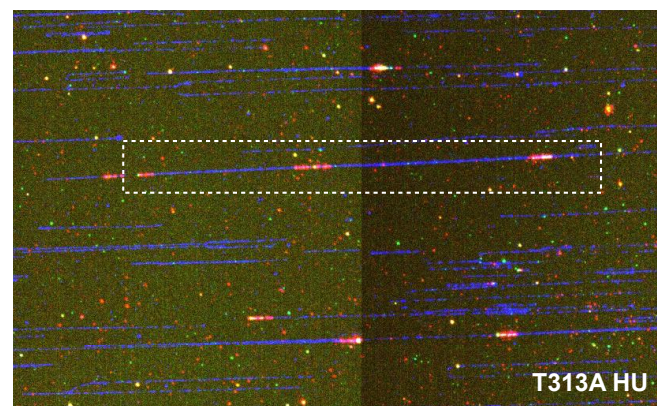

100kb

Supplement: Supplementary file 3. — The field in white squares are region presented in Figure 5B. [file elife-29953-supp3.pdf]
